# Intractable hiccups as the presenting symptom of toxic nodular goiter

**DOI:** 10.1016/j.pbj.0000000000000019

**Published:** 2018-07-03

**Authors:** Luís Miguel Fernandes Teles, Inês Domingues Neto, Bernardo Luís Fernandes Macedo, Fernando Montira

**Affiliations:** Internal Medicine Department, Hospital São Sebastião, Centro Hospitalar Entre Douro e Vouga, Santa Maria da Feira, Portugal.

**Keywords:** goiter, hiccups, hyperthyroidism

## Abstract

Hiccups differential diagnosis is a challenging one often being inconclusive and sometimes attributed to malignancies, and so of extreme importance to an internist. Seventy-five-year-old man with history of alcohol abuse, hypertension, and hyperlipidemia presented to the emergency department after having initiated diarrhea, hiccups, and vomiting for 4 days. Physical examination revealed signs of dehydration and persistent hiccups at rest. Laboratory investigations revealed acute renal failure (creatinine 3.7 mg/dl, reference value: 0.7–1.3 mg/dl; urea 195 mg/dl, reference value: 18–55 mg/dl) and no elevation of inflammatory parameters. Findings were consistent with a gastroenteritis, it was started fluids and the patient was admitted in the internal medicine ward. As the gastroenteritis symptoms ceased and the acute renal failure was resolved, the hiccups continued and physical examination revealed 2 palpable thyroid nodules. Laboratory findings shew subclinical hyperthyroidism (serum TSH 0.02 uUI/ml, reference value: 0.35 –4.94 uUI/ml; free T4 levels 18.5 pmol/L, reference value: 9.0–19 pmol/L). It was conducted an ultrasonography that revealed an increase of thyroid dimensions and 2 nodules. One nodule in the right lobe with 32 mm of dimension and one nodule in the left lobe with 58 mm of dimension. Both nodules were hypoechoic. Patient started antithyroid medication with propylthiouracil (PTU), 200 mg every 12 hours, and a cervical CT scan was conducted. CT scan revealed images compatible with diving goiter (Fig. 1) and tracheal deviation, for the right side (Fig. 2), inducted by the thyroid left nodule. Patient was discharged with antithyroid medication and hiccups were meliorated with chlorpromazine although persisting. After thyroid function normalization thyroidectomy was conducted, a few months later, and hiccups ceased.

Hiccups are a common and usually transient condition affecting almost all people in their lifetime. Rarely, hiccups become intractable and can lead to adverse outcomes. Hiccups can lead to significant adverse outcomes including malnutrition, weight loss, fatigue, dehydration, insomnia, mental stress, and decreased quality of life.^1^ Hiccups differential diagnosis is a challenging one often being inconclusive and sometimes attributed to malignancies, and so of extreme importance to an internist.

Authors report a 75-year-old man with a history of alcohol abuse, hypertension, and hyperlipidemia that presented to the emergency department after having initiated diarrhea, hiccups, and vomiting for 4 days. Physical examination revealed signs of dehydration and persistent hiccups at rest. Laboratory investigations revealed acute renal failure (creatinine 3.7 mg/dl, reference value: 0.7–1.3 mg/dl; urea 195 mg/dl, reference value: 18–55 mg/dl) and no elevation of inflammatory parameters. These findings were consistent with a gastroenteritis, it was started fluids and the patient was admitted in the internal medicine ward. After 2 days, hiccups continued and the patient started chlorpromazine 25 mg every 8 hours. As the gastroenteritis symptoms ceased and the acute renal failure was resolved, the hiccups continued. Several of the conditions associated with persistent and intractable hiccups can be diagnosed by a thorough history and physical examination. The persistence of hiccups during sleep suggests an organic rather than psychogenic etiology.^2^

After a careful evaluation of the patient physical examination revealed 2 palpable thyroid nodules. Laboratory findings revealed subclinical hyperthyroidism (serum TSH 0.02 uUI/ml, reference value: 0.35–4.94 uUI/ml; free T4 levels 18.5 pmol/L, reference value: 9.0–19 pmol/L). It was conducted an ultrasonography that revealed an increase of thyroid dimensions and 2 nodules. One nodule in the right lobe with 32 mm of dimension and one nodule in the left lobe with 58 mm of dimension. Both nodules were hypoechoic. Patient started antithyroid medication with PTU, 200 mg every 12 hours, and a CT scan was conducted. Chest computed tomography (CT) scan is helpful to detect pulmonary and mediastinal abnormalities irritating the vagal or phrenic nerves or the diaphragm in patients presenting with hiccups. Cervical CT scan revealed images compatible with diving goiter (Fig. 1) and tracheal deviation, for the right side (Fig. 2), inducted by the thyroid left nodule. This deviation could be inducing abnormal excitation of the phrenic nerves leading to intractable hiccups. Patient was discharged with antithyroid medication and hiccups were meliorated with chlorpromazine although persisting. After thyroid function normalization thyroidectomy was conducted, a few months later, and hiccups ceased.

**Figure 1:**
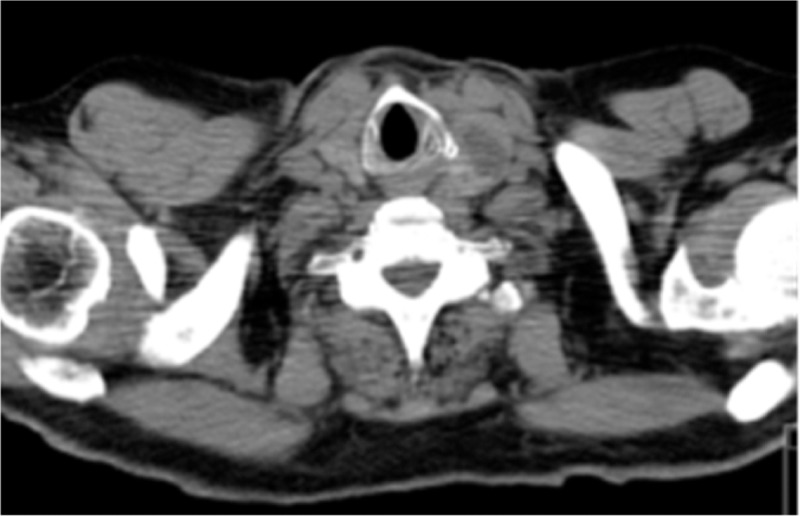
CT scan revealing diving goiter. Detailed head and neck examination is important to exclude an enlarged thyroid. Goiter was confirmed by CT scan particularly an enlarged left thyroid lobe.

**Figure 2:**
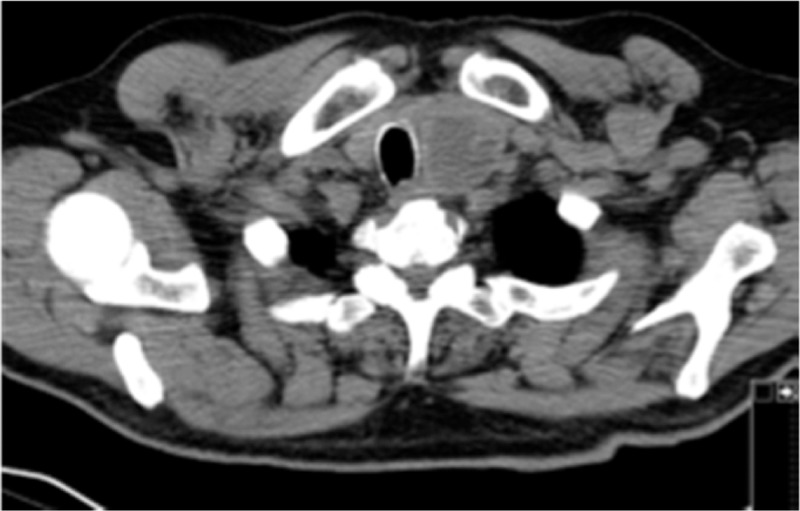
CT scan revealing tracheal deviation. Right tracheal deviation as seen in the figure could have led to irritation of the phrenic nerve promoting hiccups.

Goiters, tumors, or cysts in the neck, mediastinal masses, and abnormalities of the diaphragm that irritate the phrenic nerve are common causes of persistent or intractable hiccups, so it was mandatory to exclude them in this case report.^3^ The exact mechanism provoking hiccups remains unknown.^4^ Toxic nodular goiter is a common cause of hyperthyroidism, second in prevalence only to Grave disease.^5^ The prevalence of toxic nodular goiter increases with age and in the presence of iodine deficiency. Although the introduction of iodized salt has eliminated many cases of goiter it still is very common and patients with long-standing goiters may develop symptoms of obstruction due to progressive compression of the trachea and phrenic nerve paralysis due to obstructive goiter.

## Acknowledgments

None.

## Conflicts of interest

The authors declare no conflicts of interest.

## Abbreviations

Abbreviations: CT = computed tomography, free t4 = free thyroxine, PTU = propylthiouracil.
